# The Use of Heroin

**Published:** 1907-07

**Authors:** 


### THE USE OF HEROIN.

A Crha gives the following excellent description of the action of heroin.

Heroin is especially beneficial in the treatment of cough. A dose of .003 gram given at night to patients kept awake by cough insures ease for from four to eight hours, and the cough next day is found to be looser than before. In advanced cases of subjective symptoms of distressed breathing and pressure on the chest are relieved as effectually as by morphine, and in some cases the breathing becomes slower and inspiration more prolonged. No effect is produced on night sweats. In laryngeal tuberculosis the drug is of great benefit. The troublesome cough disappears after its prolonged use. In one case where the pain in swallowing was such that cocaine had to be applied several times a day applications twice a day of a 1 in 30 solution of heroin afforded relief. In hæmoptysis heroin replaces morphine, and in no case causes the vomiting which is so frequent after morphine. The drug has no harmful effect on the alimentary tract, and, indeed, the dyspepsia of phthisical patients has often improved rapidly when heroin was given for the cough. Crha has employed heroin in acute and chronic bronchitis, and has found it a good substitute for the usual narcotics. It diminishes the cough and lessens the pain of breathing. The distressed breathing of emphysematous patients when dependent on heart disease is relieved even before rest and heart stimulants have had time to act. In one case of cardiac asthma the customary nightly attack was prevented by a dose of 0.005 gram of heroin. There is no effect on the heart's rhythm. As an analgesic heroin is not of much benefit except in diseases of the genital organs. Thus tampons steeped in glycerinated solution of heorin (1.5 in 1000) quickly relieve the pain of an early perimetritis. They have less effect in chronic perimetritis. As a hypnotic heroin has no direct action. It may be injected instead of morphine before giving ether as a geneal anæsthetic. It will then lessen the stage of excitement, hasten the narcosis, and prevent post-narcotic vomiting. A great advantage which heroin possesses over earlier norcotics is that only small doses are needed. Six to eight drops of a 1 per cent. solution of heroin operate as effectually as 15 drops of 2 per cent. codein, and 3 to 5 gm. of heorin as 0.01 gram of morphine. Moreover, it is only after long use that the dose of heorin needs to be increased to any appreciable extent. Heroin can be safely given to old people when morphine would be dangerous from its effect on the heart. British Med. Journal.

